# Reducing the price of treatment for multidrug-resistant tuberculosis through the Global Drug Facility

**DOI:** 10.2471/BLT.14.145920

**Published:** 2015-02-27

**Authors:** Kaspars Lunte, Thierry Cordier-Lassalle, Joel Keravec

**Affiliations:** a Stop TB Partnership, Chemin de Blandonnet 2, 1214 Geneva, Switzerland.; b World Health Organization, Geneva, Switzerland.

## Abstract

**Problem:**

Many countries have limited experience of securing the best prices for drugs and have little negotiating power. This is particularly true for the complex, lengthy and expensive regimens used to treat multidrug-resistant tuberculosis.

**Approach:**

The Stop TB Partnership’s Global Drug Facility is dedicated to improving worldwide access to antituberculosis medicines and diagnostic techniques that meet international quality standards.

**Local setting:**

The Global Drug Facility is able to secure price reductions through competitive tendering among prequalified drug manufacturers and by consolidating orders to achieve large purchase volumes. Consolidating the market in this way increases the incentives for suppliers of quality-assured medicines.

**Relevant changes:**

In 2013 the Global Drug Facility reduced the price of the second-line drugs it supplies for multidrug-resistant tuberculosis: the overall cost of the longest and most expensive treatment regimen for a patient decreased by 26% – from 7890 United States dollars (US$) in 2011 to US$ 5822 in 2013.

**Lessons learnt:**

The price of treatment for multidrug-resistant tuberculosis supplied by the Global Drug Facility was reduced by consolidating orders to achieve large purchase volumes, by international, competitive bidding and by the existence of donor-funded medicine stockpiles. The rise in the number of suppliers of internationally quality-assured drugs was also important. The savings achieved from lower drug costs could be used to increase the number of patients on high-quality treatment.

## ملخص

**المشكلة:**

تعاني بلدان عديدة من محدودية خبرات تأمين أفضل الأسعار للأدوية وليس لديها سوى صلاحيات تفاوض قليلة. وينطبق هذا بوجه خاص على نظم العلاج المعقدة والطويلة وباهظة الثمن التي تستخدم لعلاج السل المقاوم للأدوية المتعددة.

**الأسلوب:**

يتخصص مرفق الأدوية العالمي التابع لشراكة دحر السل في تحسين الوصول على الصعيد العالمي إلى الأدوية المضادة للسل وتقنيات التشخيص التي تلبي معايير الجودة الدولية.

**المواقع المحلية:**

يستطيع مرفق الأدوية العالمي تأمين انخفاضات في الأسعار من خلال إجراء مناقصات تنافسية بين صانعي الأدوية المؤهلين مسبقاً وعن طريق تعزيز الطلبات لتحقيق أحجام شراء ضخمة. ويزيد دمج السوق بهذه الطريقة الحوافز لموردي الأدوية مضمونة الجودة.

**التغيّرات ذات الصلة:**

في عام 2013، قام مرفق الأدوية العالمي بخفض أسعار أدوية الخط الثاني التي يقوم بتوريدها لمكافحة السل المقاوم للأدوية المتعددة: وانخفضت التكلفة الإجمالية لأطول نظم العلاج وأبهظها ثمناً للمريض بمقدار 26 % - أي من 7890 دولاراً أمريكياً في عام 2011 إلى 5822 دولاراً أمريكياً في عام 2013.

**الدروس المستفادة:**

تم خفض أسعار علاج السل المقاوم للأدوية المتعددة الذي يقوم بتوريده مرفق الأدوية العالمي عن طريق دمج الطلبات لتحقيق أحجام شراء ضخمة، عن طريق إجراء مناقصات دولية تنافسية وعن طريق إنشاء مخزونات احتياطية من الأدوية الممولة من المانحين. وكان الارتفاع في عدد موردي الأدوية مضمونة الجودة على الصعيد الدولي مهماً كذلك. ويمكن استخدام الوفورات الناتجة عن خفض تكاليف الأدوية لزيادة عدد المرضى الذين يتلقون العلاج عالي الجودة.

## 摘要

**问题:**

许多国家在制定最佳药物价格上经验有限，几乎没有谈判权。对于复杂、漫长且昂贵的多耐药性肺结核疗程来说尤其如此。

**方法:**

遏制结核病合作关系全球药物机构（The Stop TB Partnership’s Global Drug Facility）致力于改善全球对符合国际质量标准的抗痨药物和诊断技术的使用。

**当地状况:**

全球药物机构能够通过在具有资格的药物制造商间竞标并借助合并订单实现大量购买来确保药物降价。以这种方法整合市场提高了优质药物供应商们的积极性。

**相关变化:**

2013年，全球药物机构降低了供应给多耐药性肺结核病的二线药物价格。肺结核病人耗时最长、最昂贵的治疗总费用降低了26%，从2011年的7890美元降至2013年的5822美元。

**经验教训:**

通过合并订单实现的大量购买、国际竞标以及捐助者资助的药物库存，全球药物机构降低了其供应的多耐药性肺结核药物的价格。国际优质药物供应商数量的增加也起了重要作用。药物成本降低节省下来的资金有助于让更多病人获得高质量的治疗。

## Résumé

**Problème:**

De nombreux pays ont peu d'expérience dans l'obtention des meilleurs prix pour les médicaments et sont en position de faiblesse pour négocier. Cela est particulièrement vrai pour les traitements complexes, longs et coûteux qui sont utilisés pour traiter la tuberculose multirésistante aux médicaments.

**Approche:**

Le Dispositif mondial d'approvisionnement en médicaments du partenariat Stop TB est dédié à l'amélioration dans le monde de l'accès aux médicaments antituberculeux et aux techniques de diagnostic qui répondent aux normes de qualité internationales.

**Environnement local:**

Le Dispositif mondial d'approvisionnement en médicaments est capable de garantir des réductions de prix via des appels d'offre compétitifs lancés auprès des fabricants de médicaments pré-qualifiés et via le regroupement des commandes pour arriver à de grands volumes d'achat. Cette manière de procéder à des achats groupés augmente les incitations aux fournisseurs pour qu'ils produisent des médicaments de qualité garantie.

**Changements significatifs:**

En 2013, le Dispositif mondial d'approvisionnement en médicaments a réduit le prix des médicaments de deuxième intention qu'il fournit pour la tuberculose multirésistante aux médicaments: le coût global du protocole thérapeutique le plus long et le plus coûteux a diminué de 26% – de 7890 dollars des États-Unis d'Amérique (US$) en 2011 à 5822 US$ en 2013.

**Leçons tirées:**

Le prix du traitement pour la tuberculose multirésistante aux médicaments fourni par le Dispositif mondial d'approvisionnement en médicaments a été réduit par les achats groupés pour parvenir à de grand volumes d'achats, par les appels d'offre internationaux et compétitifs, et par l'existence de réserves de médicaments financés par les donateurs. La hausse du nombre de fournisseurs de médicaments de qualité garantie dans le monde a également été importante. Les économies réalisées grâce à la baisse des coûts des médicaments pourraient être utilisées pour augmenter le nombre de patients bénéficiant de traitement de qualité élevée.

## Резюме

**Проблема:**

Многие страны имеют небольшой опыт обеспечения минимальных цен на лекарственные препараты и ограниченные возможности ведения переговоров. Это особенно верно, когда речь идет о сложных, продолжительных и дорогостоящих схемах приема лекарств, применяющихся при туберкулезе с множественной лекарственной устойчивостью.

**Подход:**

Глобальный механизм по обеспечению лекарственными средствами Партнерства «Остановить туберкулез» предназначается для расширения доступа к противотуберкулезным лекарственным препаратам и соответствующим международным стандартам качества методам диагностики во всем мире.

**Местные условия:**

Глобальный механизм по обеспечению лекарственными средствами позволяет обеспечить снижение цен за счет конкурсных закупок у прошедших предварительную проверку производителей лекарственных препаратов и объединенных заказов, увеличивающих объемы закупок. Такое консолидирование рынка более эффективно стимулирует поставщиков лекарственных средств гарантированного качества.

**Осуществленные перемены:**

В 2013 г. Глобальный механизм по обеспечению лекарственными средствами позволил снизить стоимость лекарственных препаратов второй линии, поставляемых для лечения туберкулеза с множественной лекарственной устойчивостью: общая стоимость наиболее продолжительной и дорогостоящей схемы приема лекарств для одного пациента снизилась на 26% — c 7 890 долларов США в 2011 г. до 5 822 долларов США в 2013 г.

**Выводы:**

Стоимость препаратов для лечения туберкулеза с множественной лекарственной устойчивостью, поставляемых при помощи Глобального механизма по обеспечению лекарственными средствами, снизилась за счет составления объединенных заказов, увеличивающих объемы закупок, проведения международных конкурсных торгов и наличия запасов лекарственных препаратов, приобретенных благодаря спонсорскому финансированию. Кроме того, важную роль сыграл рост количества проверенных поставщиков лекарственных препаратов, соответствующих международным стандартам качества. Средства, сэкономленные благодаря снижению стоимости лекарственных препаратов, могут быть использованы для увеличения количества пациентов, получающих высококачественное лечение.

## Resumen

**Situación:**

Muchos países tienen una experiencia limitada en garantizar los mejores precios de medicamentos y poco poder de negociación, lo cual es particularmente cierto en el caso de los regímenes complejos, largos y costosos utilizados para tratar la tuberculosis multirresistente.

**Enfoque:**

La asociación Stop TB del Servicio Farmacéutico Mundial se dedica a mejorar el acceso a nivel mundial a los medicamentos antituberculosos y las técnicas de diagnóstico que cumplen con los estándares internacionales de calidad.

**Marco regional:**

El Servicio Farmacéutico Mundial es capaz de lograr reducciones de precios mediante la licitación competitiva entre fabricantes de medicamentos precalificados y la consolidación de pedidos para lograr grandes volúmenes de compra. Consolidar el mercado de esta manera aumenta los incentivos para los proveedores de medicamentos con garantía de calidad.

**Cambios importantes:**

En 2013, el Servicio Farmacéutico Mundial redujo el precio de los medicamentos de segunda línea que suministra para la tuberculosis multirresistente: el coste total del régimen de tratamiento más largo y más caro para un paciente disminuyó un 26 % – de 7890 dólares de Estados Unidos (US$) en el 2011 a US$ 5822 en 2013.

**Lecciones aprendidas:**

El precio del tratamiento para la tuberculosis multirresistente suministrado por el Servicio Farmacéutico Mundial se redujo mediante la consolidación de pedidos a fin de comprar grandes volúmenes, la licitación internacional competitiva y la existencia de arsenales de medicina financiados por donantes. También fue importante el aumento del número de proveedores de medicamentos con garantía de calidad internacional. Los ahorros obtenidos al disminuir los costes de medicamentos podrían aprovecharse para aumentar el número de pacientes que reciben un tratamiento de alta calidad.

## Introduction

Tuberculosis remains a major global public health problem. According to a 2014 report from the World Health Organization (WHO), only 97 000 patients of the estimated 300 000 patients with multidrug-resistant tuberculosis worldwide were receiving treatment.^1^ Access to quality medicines for patients in need is restricted by the limited availability of funding, which is often compounded by poor knowledge of drug management (e.g. storage and distribution) and a lack of staff and facilities. To increase cure rates, it is important that antituberculosis medicines are affordable and that systems are in place for providing proper care at all levels.

Many countries have limited experience in securing the best possible prices for drugs and have little negotiating power since they are not able to consolidate purchases into large volumes. This is especially true of the medicines needed for multidrug-resistant tuberculosis, where treatment is complex and can last two years or more. Moreover, these medicines are much more expensive than those for drug-sensitive tuberculosis.^2^^,^^3^

The Global Plan to Stop Tuberculosis, which was launched by the Stop TB Partnership, identified universal access to high-quality care for all people with the disease as one of its central objectives.^4^ Today, access to quality-assured drugs is promoted by key stakeholders such as the WHO Prequalification Programme,^5^ the Global Fund to Fight AIDS, Tuberculosis and Malaria, UNITAID and the Global Drug Facility, which was established by the Stop TB Partnership.

## Global Drug Facility

The Global Drug Facility is dedicated to improving access worldwide to tuberculosis medicines and diagnostic techniques that meet international quality standards. In practice, the facility provides only internationally quality-assured medicines that are manufactured under stringent conditions so that countries and their governments can be confident they will always receive high-quality medicines. This stringency ensures that risk of developing drug-resistance is minimized. Recent studies show that the substandard and falsified drugs readily available on the private market have probably contributed to the development of antituberculosis drug-resistance in low- and middle-income countries.^6^^,^^7^

Today a growing number of antituberculosis medicines are able to meet international quality standards, as verified by the WHO Prequalification Programme or other stringent drug regulatory authorities. In this context, the Global Drug Facility has contributed significantly to drug volume consolidation and has, over the years, consistently secured lower prices for quality-assured antituberculosis medicines.^8^

## Price reductions

**Fig. 1:**
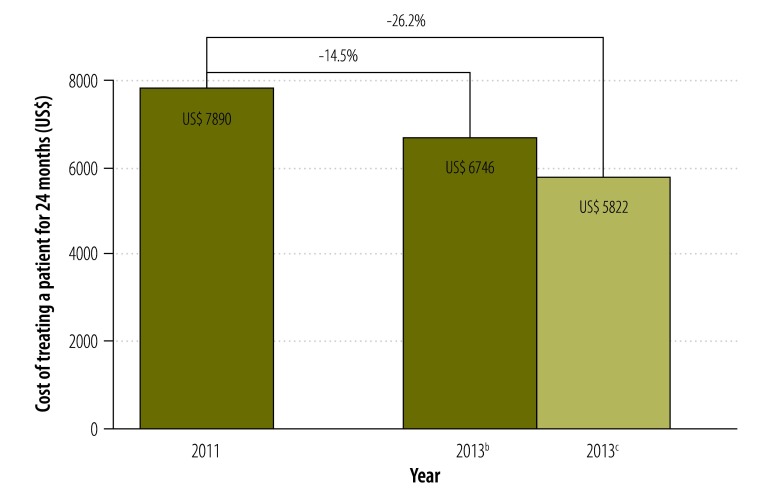
Cost of selected treatment^a^ for multidrug-resistant tuberculosis from the Global Drug Facility, 2011–2013

In 2013, as in previous years, the Global Drug Facility reduced the price of the second-line drugs it supplies for the treatment of multidrug-resistant tuberculosis. This has resulted in a significant decrease in the overall cost of treatment. Fig. 1 illustrates the change between 2011 and 2013 in the cost of the longest and most expensive regimen for treating multidrug-resistant tuberculosis, one of many regimens available worldwide. For a 24-month treatment course, the cost of treating one patient decreased by up to 26% – from 7890 United States dollars (US$) to US$ 5822 – over this period. In calculating costs, we used nominal prices obtained from the Global Drug Facility and did not adjust for either inflation or exchange rates.

US$; United States dollars.

^a^ The selected treatment was the longest and most expensive regimen for multidrug-resistant tuberculosis: 12 months of capreomycin, protionamide, cycloserine, moxifloxacin and *para*-aminosalicylic acid sodium salt, followed by 12 months of protionamide, cycloserine, moxifloxacin and *para*-aminosalicylic acid sodium salt.

^b^ Cost of treatment from the same suppliers as in 2011.

^c^ The lowest-cost treatment in 2013.

The price reductions obtained by the Global Drug Facility were secured through a competitive and transparent tendering process among the manufacturers of prequalified, antituberculosis drugs and by the facility’s continuing efforts to consolidate orders. During this time, the number of suppliers of quality-assured drugs for multidrug-resistant tuberculosis has increased. In 2012, a capacity assessment carried out by the Global Drug Facility found that a greater number of manufacturers were now able to supply internationally quality-assured, second-line drugs for multidrug-resistant tuberculosis and that, as a result, production capacity could, if required, be rapidly expanded to satisfy twice the current demand.

The actions of the Global Drug Facility have also led to an increase in the number of courses of treatment for multidrug-resistant tuberculosis delivered. In 2013, the facility delivered a sufficient quantity of various drug combinations to provide 32 000 courses of treatment, compared with 19 600 courses in 2011.

## Discussion

A summary of the main lessons learnt from the operation of the Global Drug Facility is given in Box 1. First, the expansion of the supplier base for internationally quality-assured, second-line drugs for multidrug-resistant tuberculosis ensures competition in the drug market that enabled the Global Drug Facility to consistently secure low prices. Second, the ability of the Global Drug Facility to increase the volume of drug purchases by consolidating orders from different purchasers also contributed to lower costs, as did the system of competitive bidding involving long-term agreements and the existence of the donor-funded rotating stockpile. The stockpile also helped decrease delivery times. Third, the resulting drug cost savings led to an increase in the number of courses of treatment delivered. In the future, these savings could be used by governments and donors to further increase the number of patients treated, which could, in turn, contribute to even greater consolidation of orders and, hence, to additional reductions in the cost of quality-assured drugs.

Box 1 **Summary of main lessons learnt**

- The increase in the number of suppliers of internationally quality-assured, second-line drugs for multidrug-resistant tuberculosis provided the competition needed for the Global Drug Facility to secure consistently low prices.
- The price of drugs supplied by the Global Drug Facility was reduced by: (i) consolidating orders to achieve large purchase volumes; (ii) transparent, international, competitive bidding; and (iii) medicine stockpiles funded by donors.
- The savings achieved from the lower cost of high-quality medicines can be used to increase the number of patients treated.
